# Artesian Wells

**Published:** 1872-01

**Authors:** W. L. Lipscomb

**Affiliations:** Columbus, Miss.


### THE GEORGIA MEDICAL COMPANION:

A MONTHLY ADVISER.

VOL. 2. ATLANTA, GA., JANUARY, 1872. NO. 1

### PART I.

### Original Communications and Special Selections.

### ARTESIAN WELLS.

BY W. L. LIPSCOMB, M. D., COLUMBUS, MISS.

The well from which the name *Artesian* is derived, was bored in the province of Artois, in France, in the year 1126, and has flowed steadily ever since. The water rises eleven feet above the ground and supplies nearly 250 gallons per minute.

The term *Artesian* is applied only to those bored wells, the water of which rises above the surface of the ground in a continued stream, and without artificial means. Artesian wells have been in use in France, Italy and probably in China, for several centuries. They were introduced into Germany and England about 70 or 80 years ago, and into the United States since 1840.

The first records concerning Artesian wells, were in an Italian work published in 1691, and in a subsequent one published in 1729.

It is supposed that some of the distinguished wells mentioned in the Bible were bored wells, probably Artesian, and date back to the earliest patriarchal and historical times. Wells are spoken of in Egypt 500 years after Christ, from 600 to 1,000 feet deep, that sent forth water like rivers, and were used to irrigate the country.

Among the most distinguished Artesian wells of the present day are the following, viz : *Grenelle Well*, at Paris, begun in 1834, and completed in 1841, which is 1,806 feet deep, and has a steady flow of water of 500,000 gallons in twenty-four hours. The *Kessinger Well*, in Bavaria, 1,878 feet deep, from which 100 cubic feet of water gushes every minute. An Artesian well at Bourm, in England, only 92 feet deep delivers 557,000 gallons of water per day, and rises 30 feet 9 inches above ground.

In 1858, a French engineer bored an Artesian well in the District of *Sahara*, *Africa*, and procured a jet of water of about 1,000 gallons a minute; since then, four other wells have been bored in the desert, yielding enormous quantities of cool, delicious water.

In the United States the most celebrated wells are, viz : one at St. Louis, commenced in 1849 and completed in 1854, at a cost of $10,000. It is 2,199 feet deep, and yields about 75 gallons of water per minute. Another, at Louisville, bored in 1857 and 1858, 2,086 feet deep. The water will rise in a jet 170 feet above the surface, and delivers 330,000 gallons of water in twenty-four hours. Another, at Charleston, South Carolina, 1,800 feet deep, and yields 30,000 gallons in twenty-four hours.

Artesian wells were bored in Alabama and Mississippi early after 1840, and by 1850, were quite common in all the counties in those States, lying upon the Tom Bigbee river. The most northerly Artesian well being three miles north of Camargo—depth, 200 feet, and rising two feet above the surface. At Aberdeen, the public well is 512 feet deep. At Columbus, 371 feet, and at Macon, 760 feet. On the east bank of the river the wells vary in depth from 150 to 320 feet, while on the western side, they are much deeper, from 500 to 800 feet, owing to the dip and irregularity of the water-bearing stratification. The quantity of water flowing out from these wells varies from two or three gallons to 360 gallons per minute—18 or 20 feet being the maximum height to which the water has been made to rise. The temperature of the water increases according to the depth of the well, at the rate of 1° Fhr. to every 55 feet of depth. The shallower the well the cooler the water.

Artesian wells depend upon the internal constitution and arrangement of the different strata of the earth, and therefore cannot be bored everywhere. Certain different geological strata must alternate with each other. There must be a water-bearing stratum, which is usually composed of loose material, such as sand, gravel, pebbles, etc., situated between two strata, impervious to water, which are generally composed of tenacious clays or seams of rock. These three strata must not lie horizontally, but must have a considerable dip or inclination to one side, so that water falling upon the upper surface of the water-bearing stratum will percolate to the bottom of the dip and perhaps accumulate in large pools or saturate the entire water stratum with water. The atmosphere and the height of the water above make great pressure upon the water below, and by the hydrostatic law "that water will rise as high as it falls," a well bored into this stratum, gives an outlet to the water, which, in the effort to produce an equilibrium, rises above the surface of the ground.

The geological formations which underlie the counties of Lowndes and Octibbihaw, particularly upon the western side of the Tom Bigbee river, are unusually favorable in the particulars above mentioned.

The Etawa and Tom Bigbee sand group—the lower group of the Cretaceous formation, consists mostly, of green sands, which are seperated into different strata by seams of indurate sand cemented by lime and silica, or of seams of the oxide of iron mixed with sand. This arrangement makes them very prolific water bearers and adapted to the establishment of Artesian wells.

The strata in these localities have also a considerable dip—the highest portion of which is found upon the high hills on Buttahatchie and Luxapalila rivers, an altitude about 400 feet above the ocean, while Columbus and other high points are only about 300 feet—the bottoms and level lands being lower still, thus presenting the necessary elevation on one side and depression on the other. This dip or inclination extends in a south-west direction from Columbus, at the rate of about twenty-five feet per mile, and Artesian wells south-west of Columbus, have to increase in depth at that rate.

Hundreds of Artesian wells have been successfully bored, beginning at the bases of the Buttahatchie and Luxapalila hills and extending throughout the bottoms and second bottoms of Lowndes and Pickens and Greene counties to the Tom Bigbee river, at a depth varying from 150 to 500 feet.

The water of these wells will next claim consideration. The Etawa group of the cretaceous formation gives origin to a very inferior variety of mineral water, viz : the Chalybeate. This is not at all surprising, considering the nature of the formation itself —which consists of rocks and sands, and clays, composed of the *Silicate* of Iron, which give to them their characteristic hue, and also name of Glancarite or Green sand. These sands are seperated into layers or penetrated by seams and veins of the *oxide of Iron*—while diffused throughout, are quantities of rounded balls of various sizes, called *iron* pyrites or sulphur balls, composed of the *Sulphuret of Iron.*

The Chalybeate waters are produced from the disintegration and solution of those various ferruginous combinations, and take their variety from the combination most involved.

The Iron Pyrites or Sulphur balls—from their non-soluble character, furnishes the largest proportion of the Chalybeate water, and is generally as copperas water.

Some of the wells—like Dr. Rabb's in Columbus, show a large impregnation of the Silicate of Iron—others taste of Lime or Magnesia and Soda, but these are dependent, no doubt, upon their passage through strata or soils containing those ingredients.

In some of the Chalybeate wells, reached at the shallowest depths, we find a preponderance of the *Sulphur* over the Iron, readily recognized by the emission of Sulphuretted-hydrogen gasses and the sulphurous taste, and the further fact that they are good, soft waters, useful for washing purposes, that bleach well and are free from the copperas coloring property. These wells are regarded as fine drinking waters, of a cool temperature, about 40 deg., and are highly prized by the residents. They are usually attainable at about 150feet depth. but generally in small streams, and are liable to cessation.

The deeper and fewer wells are all strongly Chalybeate, useless for washing purposes, warm, are attended with heavy deposits of the oxydes of Iron along their courses and in vessels containing them.

The waters of all these varieties of wells are generally the drinking and cooking waters of all persons upon the premises and have superceded the ordinary wells in their localities.

The effect the boring of Artesian wells has had upon the health of the country, has been a subject of considerable inquiry and solicitude.

Intelligent citizens and physicians have long suspected their injurious effects, but so great has been the local conveniences and advantages of these wells that the community at large have been disposed to shut their eyes to any probable evils and to deny any injury therefrom.

But years of observation have produced such an array of facts that the medical faculty feel prepared to announce the conclusion, that *Artesian wells cause an increase of the prevalance and severity of malarial fevers.*

The correctness of this conclusion can hardly be denied in the sections of country north and south of Columbus, along the east bank of the Tom Bigbee river.

The military and Hamilton roads, which converge at Columbus, Lowndes county, Miss., are the oldest roads in that section of the State, and as they passed through a productive agricultural region were early settled and well improved. This section of country was never regarded sickley, even when the lands were first opened. But since 1841 or 1842, or since the introduction of Artesian wells, which are found at almost any homestead, malarial fevers have so increased in severity and prevalence that almost all the entire male population of the early settlers are *dead*, and the country is owned by a remnant of widows and by new settlers.

The increase and severity of the fevers has also been observed in Pickens and Green counties, Alabama, in which these Artesian wells abound. The Subute, Lipsey and Black Warrior counties have become more and more unhealthy, and of late years a new and virulent form of malarial disease, called Yellow-disease, or Hæmatural Intermittant Fever, extremely fatal, has been developed, and upon inquiry found to originate in the vicinity of these same Artesian wells.

This ugly disease has extended into this section of Mississippi, and with singular uniformity sticks closely to the bored well neighborhoods. In fact, so common is the occurrence that in the absence of some very obvious source of malarial poison, the physician inquires at once for an Artersian well. A conversation with a number of the members of this society, reveals that at least 7–10 of all their cases of yellow-disease, particularly the fatal ones, occurred on premises containing an Artesian well.

The same is true also, of that aggravated form of Algid Congestive fever, which prevailed at the first settlement of the country, but which had almost entirely disappeared. They are found reappearing in these Artesian well neighborhoods.

Any very sickly place, more so than the surrounding settlements, is very apt to be distinguished by the abundant overflowing well located thereon. The North-western portion of Columbus—the sickliest portion of the city—contains most bored wells, and until an Artesian well ran out in the main street of the city, chill and fever among the clerks and young men was almost unknown. Since that time they have been very prevalent.

Crossing to the west side of the river, we have only to inquire where are the Artesian wells, to find malarial fevers from January to January, and enough of yellow disease to mark the spots with their malignant blood-stains.

These are not idle fancies or hastily made observations, and an inquiry into their truth is challenged, both for the benefit of science and the good of the country.

In this paper, no pretence is set up that Artesian wells are the cause of *all* malarial fevers of the country, or that certain sections may not be unusually sickly *without* them. We do not even deny that they are located most generally in sections in which malarial fevers are the prevailing type.

We contend only that Artesian wells increase the prevalence and severity of malarial fevers, and that they are detrimental to the health of the places on which they are located.

It may not be amiss for us to inquire, in this paper, for the probable reasons why Artesian wells thus produce sickness.

There is no better established fact in pathology than that the use of certain mineral water, as a daily drink, produces certain diseases and liabilities to disease. The Magnesian Lime-stone waters of Switzerland and other portions of Europe and Asia, produce Cretinism and Goitrie.

The limestone regions of Tennessee and Kentucky have been marked by the desolating tread of the cholera, and the yellow-fever stops its ravages with the long moss of Florida and Alabama. Geologists have attributed the prevailing sickness of Mississippi to the almost universal absence of good water within the State. Magnesia, soda, lime and salt, seem to be ingredients in almost every well and spring. This unpleasant impregnation has caused the resort to cisterns and bored wells, and stands in the way of an appreciation of the facts and conclusions contained in our essay.

Mild Chalybeates have always been regarded the most healthy of all mineral waters, and are supposed to invigorate digestion, improve the blood and to perform all the functions of good tonic medicine.

Our only remark as to the use of mineral water as a daily and constant drink is that all the active ingredients of mineral waters are *medicines*, and that the habitual and protracted use of any medicine in any form, is prejudicial to vigorous health. What effect the continual use of Chalybeate water may have upon the human organism, in preparing it for the reception of malaria poison or breaking down its resistance thereto, we will not stop at this time to argue, but only propose the query. Could not the long protracted use of a solution of the sulphate of iron, produce, from over stimulation, a resultant depression like the use of alcohol upon the digestive functions, and so impair nutrition as to bring the system to its lowest power of resistence ?

Malaria, we are taught to regard as a poisonous emanation from earth, caused by heat, moisture and vegetable decomposition, and if we can find Artesian wells to be productive of these causes, the inference is fair and legitimate, that they must produce an additional amount of malaria to that caused by the locality before they were established. What ever may have been the health or sickliness of the place, water 150 or 500 feet under ground could have produced no effect, but if that water be brought to the surface and thereby is productive of heat, moisture and vegetable decomposition, the factors of malaria, then the Artesian well must make a healthy place sickly, and a sickly place sicklier.

The excessive temperature of our summer and fall months would furnish all the *heat* necessary for our argument, but we have, in addition thereto the fact that all soils colored with iron are active absorbents of the sun's rays and, thus attract to their locality an unusual amount of heat.

The moisture is present in an ever-flowing stream of water, and the large accumulation in the ponds made by them. We have also the geological fact that all ferruginous soils are very retentive of moisture, so much so that many of the low places in the fields, difficult to drain, and very unproductive, are supposed to be so on account of the iron they contain, which compacts the clay and produces *rust* in the vegetation.

Pipe clay lands abounding in Pyrites or Sulphur balls are notoriously poor and hard to drain.

Aitkins has the following paragraph in an article on the nature of malarious soils: "It is a fact that the usual localities in which paludal fevers abound are those which consist of mineral, vegetable and animal matters mixed together in such proportions and of such constituents chemically, as tend to absorb moisture and retain it, and subsequently to decompose." The affinities of iron for moisture is seen in the readiness of the metal to oxydize or rust, and of all its salts to deliquesce.

That these affinities may be further extended, may be inferred from the fact that in all the swamps and bottoms, and marshes in which Chalybeate springs abound, are found also large and active accumulations of malarial poison.

This truth is so well known that physicians forbid their patients remaining at Chalybeate springs any length of time for fear of contracting chills.

The moisture produced by these Artesian wells is further promoted by their usual location under the luxuriant shade-trees that abound in the yards of a country residence.

So we have moisture in the evaporation from a never-failing stream of water—we have it again in the extensive percolation of this highly charged mineral solution into the dry sand or earth through which they run—and again in the large and shaded ponds into which it empties, besides the moisture incident to the particular attraetion which ferruginous soils have for all atmospheres containing water.

The last feature in the production of malaria, is the decomposition of vegetable matter, and Artesian wells present to our mind all the conditions and circumstances for its large and active production.

In the first place, we have an unfailing supply of a solution of the sulphate of iron, one of the active properties of which is the destruction or decomposition of vegetable matter. Next, we have large quantities of vegetable and animal matter coming in the shape of refuse from the kitchen and yard—manures from the stable lots and privies, and the acumulated leaves of all the trees in the immediate vicinity washed by the rains, into the pond made, usually, in the lowest locality, by the stream from the well.

And thus our argument ends complete, if heat, moisture and vegetable decomposition are the active agents in the production of malaria, we have in our Artesian wells an artificial manufacture of all those agents, and situated too, right in the houses of the residents, where, day and night they can breath and drink, and absorb the noxious exhalations.

---

Read before the Columbus Medical Society, November 14th 1871, and requested for publication.

J. W. M. SHATTUCK, M. D., *Sec'y.*

